# Artificial intelligence-driven digital scribes in clinical documentation: Pilot study assessing the impact on dermatologist workflow and patient encounters

**DOI:** 10.1016/j.jdin.2024.02.009

**Published:** 2024-02-20

**Authors:** David Y. Cao, Jamie R. Silkey, Michael C. Decker, Karolyn A. Wanat

**Affiliations:** a School of Medicine, Medical College of Wisconsin, Milwaukee, Wisconsin; b Department of Orthopedic Surgery, Medical College of Wisconsin, Milwaukee, Wisconsin; c Department of Emergency Medicine, Medical College of Wisconsin, Milwaukee, Wisconsin; d Department of Dermatology, Medical College of Wisconsin, Milwaukee, Wisconsin

**Keywords:** artificial intelligence, clinical research, general dermatology

*To the Editor:* Documentation in electronic medical records (EMRs) occupies a significant portion of physicians’ days and detracts from patient interactions and satisfaction^1^; for every hour of patient-facing time, 2 hours are spent on documentation.^2^ The efficacy of artificial intelligence (AI)-driven digital scribe technologies with automatic speech recognition that aid in note creation has not been previously demonstrated in literature, but has potential to reduce documentation burdens while improving patient experiences.^3^, ^4^, ^5^ This pilot study explores the use of Dragon Ambient eXperience (DAX) (Nuance & Microsoft) as a digital scribe in an academic and community-based dermatology setting. The application was selected due to easy integration with the EMR and ability to convert encounters into specialty-specific notes. DAX is AI-driven, meaning that the software will learn to adjust notes based on clinician habits (language, formatting, etc) after a virtual scribe has dictated notes. Project was determined to be exempt from institutional review board (IRB).

Clinicians used DAX for documentation after training and completed a nonincentivized survey 30-60 days later (Supplementary Fig 1, available via Mendeley at https://data.mendeley.com/datasets/chsjkwf8th/1). Survey questions were provided by the company for comparison across specialties. Productivity metrics were gathered (time in notes per visit/week), and clinicians were stratified into low (<50% of encounters) and high utilizers (>70% of encounters). Patients also completed a survey following their visits (Supplementary Fig 2, available via Mendeley at https://data.mendeley.com/datasets/chsjkwf8th/1). Statistical analysis including independent samples t-test was used to compare variance across means between groups (SPSS, version 27.0; IBM Corp).

Twelve clinicians (10 dermatologists and 2 physician assistants) were onboarded and 10 elected to continue using DAX between February 2021 and January 2023. Quantitative data included productivity metrics and note characteristics (Table I) pre-DAX and post-DAX use for clinicians. Time spent per day in EMRs (aggregating “time in notes” and “time outside 7 am-5:30 pm”) among all utilizers decreased from 90.1 minutes preuse to 70.3 minutes postuse (*P* < .001). DAX users’ note contribution percentage decreased by nearly half (*P* < .001), but note length increased by ∼30-50 words (*P* < .05).

**Table I:** Impact of DAX on clinician productivity and note characteristics

	All users			Low utilizers (<50%)			High utilizers (>70%)		
	Mean (SD)	Mean (SD)	*P*∗	Mean (SD)	Mean (SD)	*P*∗	Mean (SD)	Mean (SD)	*P*∗
	Before DAX, *n* = 12	Using DAX *n* = 10		Before DAX, *n* = 8	Using DAX, *n* = 8		Before DAX, *n* = 2	Using DAX, *n* = 2	
Appointments per month	169.1 (35.4)	168.6 (23.9)	.35	157.3 (32.8)	147.0 (19.9)	.34	222.1 (46.3)	204.4 (36.9)	.12
Time in notes per day (min)	54.6 (7.4)	42.2 (5.1)	.003†	47.7 (6.3)	32.9 (3.6)	.002†	86.7 (8.4)	66.4 (8.7)	<.001†
Time in notes per appointment (min)	6.5 (1.2)	5.1 (0.8)	<.001†	6.5 (1.3)	4.8 (0.8)	<.001†	8.0 (0.9)	6.7 (0.9)	.01†
Time outside 7 am-5:30 pm in EMR per day (min)	35.5 (14.7)	28.1 (8.9)	.005†	39.4 (15.7)	35.2 (9.3)	.001†	14.6 (7.1)	14.8 (10.5)	.49
Note length (characters)	4412 (350)	4617 (444)	.03†	4713 (334)	4892 (546)	.03†	4826 (146)	4780 (259)	.42
Provider note contribution (%)	96.7 (4.6)	51.7 (7.4)	<.001†	96.8 (5.2)	43.2 (6.9)	<.001†	100.0 (0)	58.6 (11.2)	<.001†

*SD*, Standard Deviation.

∗ Assessed using the Independent Samples T-Test.

† *P* < .05.

Six clinicians completed the survey (60% response rate). 66.7% were satisfied with documentation turnaround time. 83.3% would be “very disappointed” if DAX was not available and felt it “significantly improved” the overall quality of experience with patients. They also noted the digital scribe saves time, increases note accuracy/detail, decreases documentation stress, and makes encounters more personable. Patients reported positive perceptions of encounters during usage (Fig 1).

**Fig 1:**
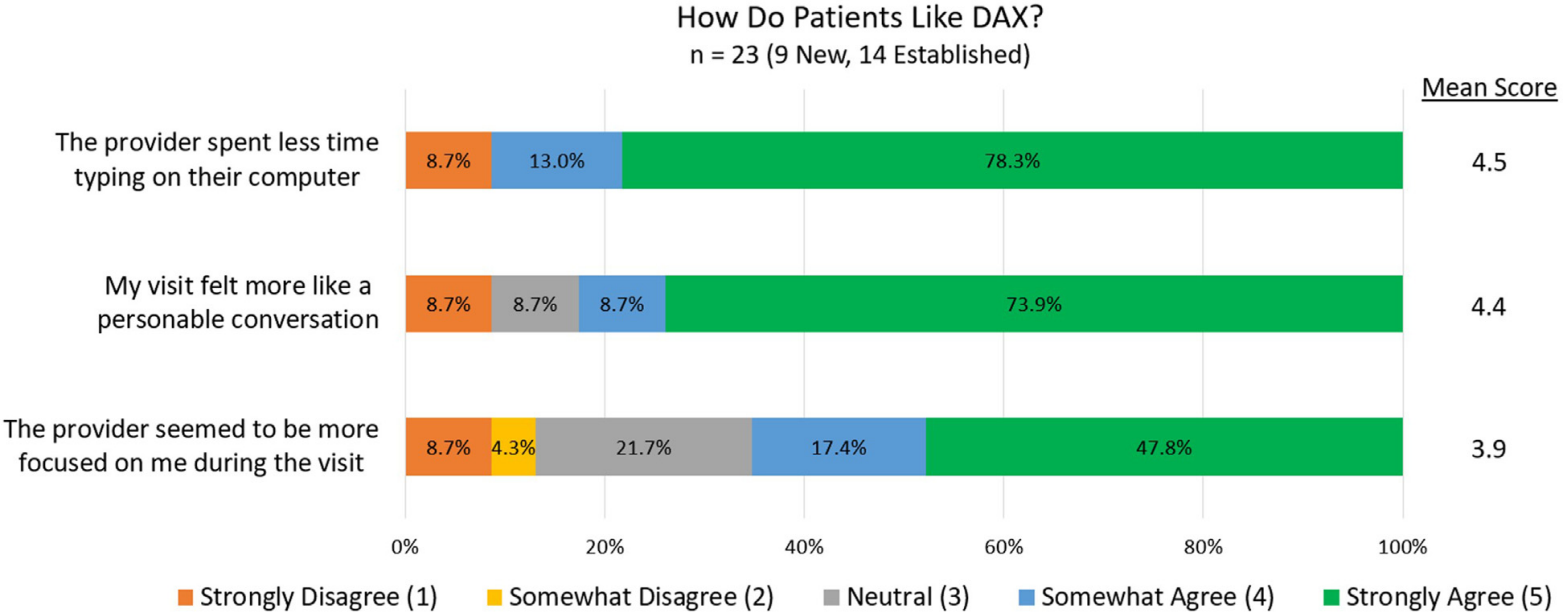
Patient perceptions of DAX. Patients were asked to rate 3 questions from 1 (strongly disagree) to 5 (strongly agree), with mean score calculated.

Compared to in-person scribes, digital scribes may confer a potential cost advantage and do not require re-training with turnover. In our study, monthly and initial software costs are $1850/month and $1000 to onboard a clinician. Locally, a scribe costs ∼$3050/month, inclusive of recruiting and training costs. Utilizing DAX over an in-person scribe could equate to ∼$13,400 to ∼$14,400 in cost-savings, although additional work that in-person scribes perform (including entering orders or patient instructions) are not possible with DAX. These costs may not be generalizable given our pilot study was performed at a large academic institution.

Limitations include the small size and single institution. Data from this pilot suggest that digital scribes decrease average documentation time, ease administrative burdens, and improve both clinician and patient experience in dermatology clinic, although other confounders could be present. Larger studies across specialty practices are needed to fully understand time and cost savings, limitations, and opportunities afforded by digital scribes. As other artificial intelligence-driven clinical documentation software is introduced, a comparison of these platforms is warranted.

## Conflicts of interest

None disclosed.
